# The flattened and needlelike leaves of the pine family (Pinaceae) share a conserved genetic network for adaxial-abaxial polarity but have diverged for photosynthetic adaptation

**DOI:** 10.1186/s12862-020-01694-5

**Published:** 2020-10-07

**Authors:** Hong Du, Jin-Hua Ran, Yuan-Yuan Feng, Xiao-Quan Wang

**Affiliations:** 1 State Key Laboratory of Systematic and Evolutionary Botany, Institute of Botany, the Chinese Academy of Sciences, 20 Nanxincun, Xiangshan, Beijing, 100093 China; 2 University of Chinese Academy of Sciences, Beijing, 100049 China

**Keywords:** Pinaceae, Leaf anatomy, Adaxial-abaxial polarity, Polarity gene, Developmental mechanism, Adaptive evolution, Photosynthesis

## Abstract

**Background:**

Leaves have highly diverse morphologies. However, with an evolutionary history of approximately 200 million years, leaves of the pine family are relatively monotonous and often collectively called “needles”, although they vary in length, width and cross-section shapes. It would be of great interest to determine whether Pinaceae leaves share similar morpho-physiological features and even consistent developmental and adaptive mechanisms.

**Results:**

Based on a detailed morpho-anatomical study of leaves from all 11 Pinaceae genera, we particularly investigated the expression patterns of adaxial-abaxial polarity genes in two types of leaves (needlelike and flattened) and compared their photosynthetic capacities. We found that the two types of leaves share conserved spatial patterning of vasculatures and genetic networks for adaxial-abaxial polarity, although they display different anatomical structures in the mesophyll tissue differentiation and distribution direction. In addition, the species with needlelike leaves exhibited better photosynthetic capacity than the species with flattened leaves.

**Conclusions:**

Our study provides the first evidence for the existence of a conserved genetic module controlling adaxial-abaxial polarity in the development of different Pinaceae leaves.

## Background

Most extant genera of conifers, the largest lineage of gymnosperms, originated in the middle and late Mesozoic, while a majority of extant species recently diverged until the Neogene and are now widely distributed throughout the world except Antarctica [1–4]. Conifers are characterized by their distinct leaves, i.e., “needles”, which underwent convergent or parallel evolution over a long history to adapt to different environments, and these leaves show two main types, needlelike and flattened leaves (including scale-like leaves). Generally, needlelike leaves appear to be more drought-resistant than flattened leaves [5, 6]. Previous studies on conifer “needles” have evaluated their morphology and physiology [7–9] but rarely focused on their developmental mechanisms and evolution. Pinaceae (the pine family), comprising 11 genera and approximately 230 species, is the most important component of the coniferous forests in the Northern Hemisphere, with both needlelike and flattened leaves [4, 10, 11]. However, it is difficult to study the molecular mechanisms underlying the evolution and development of some important traits of this family due to the large genome size, high heterozygosity and long generation time of Pinaceae [12–14].

Compared to the scarce literature on gymnosperms, leaf developmental mechanisms have been extensively studied in angiosperms. In general, angiosperm leaves develop as bifacial structures with distinct adaxial and abaxial identities, which enables the maximum photosynthetic rate [15–18]. As early as 1995, Waites and Hudson proposed a hypothesis that lamina outgrowth is promoted at the juxtaposition between adaxial and abaxial identities [19]. Then, several distinct regulators involved in leaf adaxial-abaxial polarity specification were identified, such as the adaxial determinants of the *HDZIPIII* family, *AS1-AS2* and miR165/166, and the abaxial determinants of *ETT/ARF3*, *ARF4*, the *YABBY* family and the *KANADI* family [19–25]. The miR165/166 can degrade *HD-ZIPIII* in the abaxial domain, leading to the exclusive accumulation of its transcripts in the adaxial domain [26]. The AS1-AS2 complex could repress the expression of abaxial genes, such as *KAN2*, *YAB5*, *ETT/ARF3* and *ARF4* [22, 27], and simultaneously degrade the transcripts of *ETT/ARF3* and *ARF4* by the small RNA tasiR-ARF in the adaxial domain, ultimately contributing to the determination of the adaxial cell fate [28, 29]. The mutually antagonistic interactions between the adaxial and abaxial identity regulators determine the dorsoventrally flattened structure of conventional bifacial leaves. Interestingly, a kind of unifacial leaf also exists in angiosperms, which is characterized by only an abaxialized identity usually with a radially symmetric, cylindrical structure [30–32]. In addition, the absence of the adaxial identity in bifacial leaves usually gives rise to cylindrical leaves, similar to those of unifacial leaves. Thus, it is intriguing to explore when the molecular genetic mechanism for specifying leaf adaxial-abaxial polarity originated, whether conifer “needles” are also regulated by this mechanism and whether this mechanism is conserved between the needlelike and flattened leaves in conifers. In recent years, with the development of high-throughput sequencing technologies, several genomes of gymnosperms, especially Pinaceae, have been released [12–14, 33], providing a good opportunity to disentangle how Pinaceae leaves genetically developed to different types.

Generally, variations in leaf morphology and structure are closely related to changes in physiological function, which reflect adaptations to different environments [34–36]. In gymnosperms, some previous studies indicated that the species of the pine family with needlelike leaves exhibited a higher photosynthetic rate than Podocarpaceae with flattened leaves [5, 37], which reflected that narrow or needle leaves were suited to sunny and dry environments, whereas broader leaves were adapted to typically shady and humid habitats [5, 37]. In addition, a recent study on Cupressaceae demonstrated that most species in the Cupressoid and Callitroid clades, which have appressed, imbricate leaves, were more drought-tolerant than other clades with flattened leaves [6]. Meanwhile, this study found that a relatively low photosynthetic capacity was presumably related to the small, thick-walled conduits [6]. Therefore, examination of the correlation between leaf morphology and physiological function, such as photosynthetic capacity, appears to be crucial for understanding the evolution and adaptation of Pinaceae with different types of leaves.

In this study, we investigated the developmental mechanisms and adaptive evolution of Pinaceae leaves. Based on a detailed morpho-anatomical experiment, the flattened and needlelike leaves were structurally characterized. Then, we investigated the phylogenetic relationships and expression patterns of polarity genes to test whether the developmental program for specifying leaf adaxial-abaxial polarity diverged in the two types of Pinaceae leaves. Moreover, the correlation between leaf morphology and photosynthetic capacity was examined to understand how Pinaceae have made morphological and physiological adjustments to better adapt to different habitats.

## Results

### Morphological and anatomical divergence of Pinaceae leaves

**Fig. 1:**
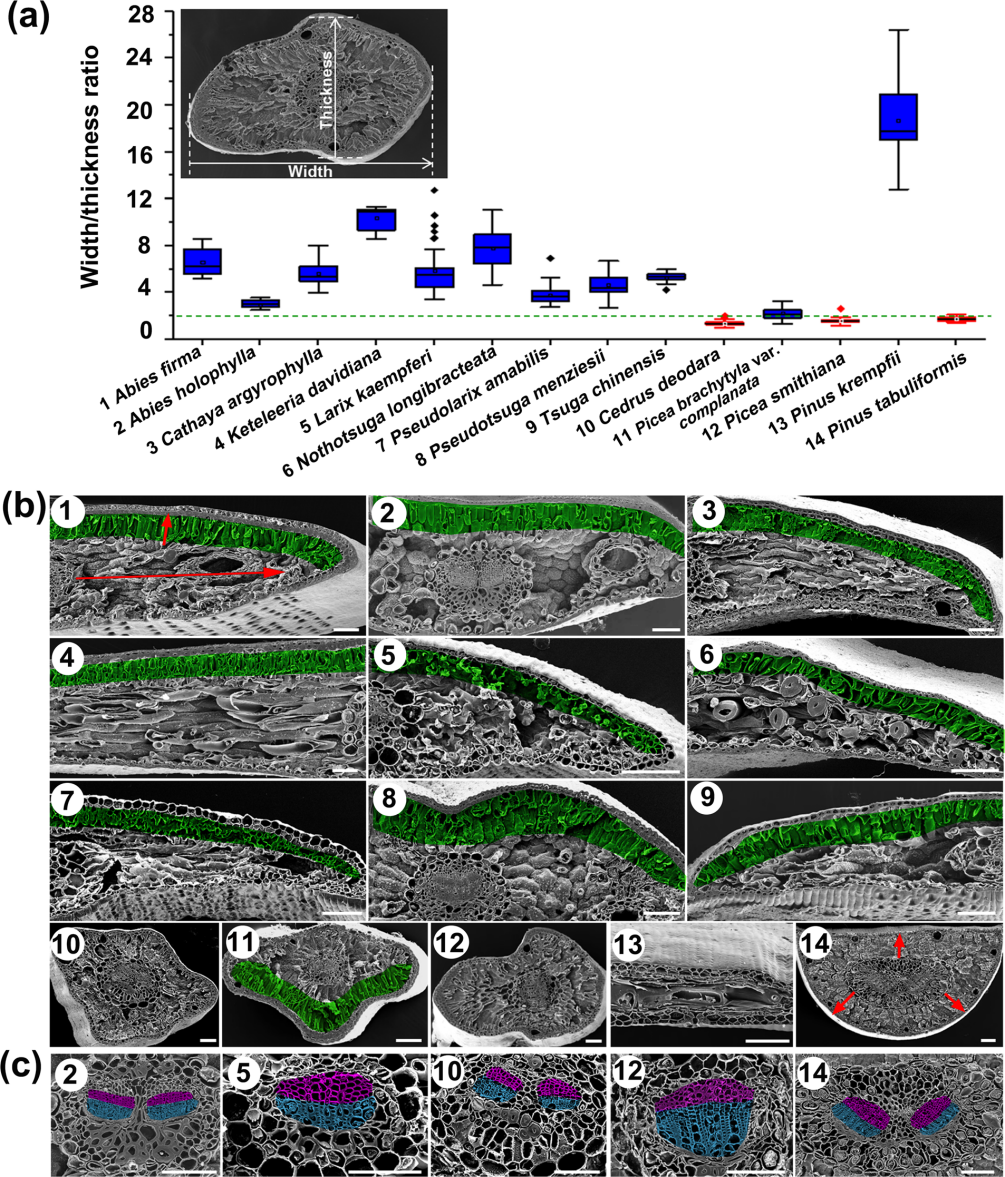
Width/thickness ratio and anatomical structures of leaf cross sections of the representative Pinaceae species. **a** Dot boxplots with an example of the width and thickness measurement shown at the top left. Red boxes, species with needlelike leaves; blue boxes, species with flattened leaves. The two boundaries of the box correspond to the 25 and 75% percentiles, respectively. The internal horizontal line and square indicate the median and mean values, respectively. **b** SEM micrographs of leaf anatomical structures of the species as shown in (**a**) with the same number, except number 9, which represents *Tsuga forrestii*. Green color, palisade tissue; red arrow, the direction of cell arrangement. Bars, 100 μm. **c** SEM micrographs of leaf vasculatures of the species as shown in (**a**) with the same number. Purple color, xylem; blue color, phloem. Bars, 100 μm

Based on the width/thickness ratio (WTR) of the cross section, Pinaceae leaves could be mainly divided into two types, needlelike (mean WTR < 2) and flattened (mean WTR > 2) (Fig. 1a and Additional file 1: Figure S1). Leaves of the selected species of *Cedrus*, *Picea* and *Pinus*, except for *Picea brachytyla* var. *complanata* and *Pinus krempfii*, belonged to the needlelike type, which were quadrangular, triangular or semicircular, and it was difficult to distinguish the adaxial or abaxial side, while samples from the remaining eight genera (*Abies*, *Cathaya*, *Keteleeria*, *Larix*, *Nothotsuga*, *Pseudolarix*, *Pseudotsuga* and *Tsuga*), as well as *Picea brachytyla* var. *complanata* and *Pinus krempfii* exhibited flattened and dorsoventrally asymmetric leaves (Fig. 1a and Additional file 1: Figure S1).

Anatomical observation by SEM indicated that the two types of leaves in Pinaceae each have distinct structural characteristics. In the flattened leaves, the mesophyll cells differentiated into asymmetric, adaxial-abaxial structures of palisade (green color) and spongy tissues (Fig. 1b, species 1–9 and 11). Two directions of mesophyll cells were uniformly aligned, with elongated palisade cells perpendicular to the adaxial surface and several layers of spongy cells along the mediolateral axis adjacent to the abaxial side (Fig. 1b, e.g., the red arrows in species 1), however, the arrangement pattern of mesophyll cells was opposite in *Picea brachytyla* var. *complanata* (Fig. 1b, species 11). Another exception was *Pinus krempfii*, which has flattened leaves, but adaxial palisade cells were not found in its leaves (Fig. 1b, species 13). In contrast, in the needlelike leaves, the mesophyll cells were generally homogeneous without obvious adaxial-abaxial divergence and radially distributed from the middle vascular bundle toward the leaf surface (Fig. 1b, species 10, 12, 14, e.g., the red arrows in species 14).

In addition, sclereids were found in three species, i.e., *Pinus krempfii*, *Pseudotsuga menziesii*, and *Nothotsuga longibracteata* (Additional file 2: Figure S2, Additional file 3: Video S1, Additional file 4: Video S2 and Additional file 5: Video S3), which are known for radially transporting water from the vein to the margin [36]. In *Pinus krempfii,* the sclereids were cylindrical and midrib-vertically, discontinuously arranged in the mesophyll (Additional file 2: Figure S2a and Additional file 3: Video S1). Likewise, the sclereids in *Pseudotsuga menziesii* were also midrib-vertical but star-shaped and connected to the parenchymatous endodermis (Additional file 2: Figure S2b and Additional file 4: Video S2). However, in *Nothotsuga longibracteata*, their sclereids were midrib-parallel, unbranched and lignified tubules, possessing thick walls, short internal diameters and long lengths comparable to the leaf length (Additional file 2: Figure S2c and Additional file 5: Video S3).

### Spatial adaxial-abaxial pattern of vasculatures in Pinaceae leaves

The spatial distribution of leaf vasculatures is important for distinguishing the adaxial-abaxial polarity of leaves [16]. One (Fig. 1c, *Larix kaempferi* and *Picea smithiana*) or two (Fig. 1c, *Abies holophylla*, *Cedrus deodara* and *Pinus tabuliformis*) vascular bundles, separated by several layers of parenchyma cells, were observed. The xylem tissues (purple color) differentiated toward the adaxial surface, while the phloem (blue color) abaxially differentiated in Pinaceae flattened leaves (Fig. 1c, *Abies holophylla* and *Larix kaempferi*). Unexpectedly, similar to the flattened leaves, the vasculatures in the needlelike leaves could also be divided into parallelly arranged xylem and phloem tissues (Fig. 1c, *Cedrus deodara*, *Picea smithiana* and *Pinus tabuliformis*). Phloroglucinol staining also confirmed the parallel xylem and phloem tissues in the needlelike leaves (Additional file 6: Figure S3ss).

### Identification and phylogenetic analysis of the polarity genes in Pinaceae

**Fig. 2:**
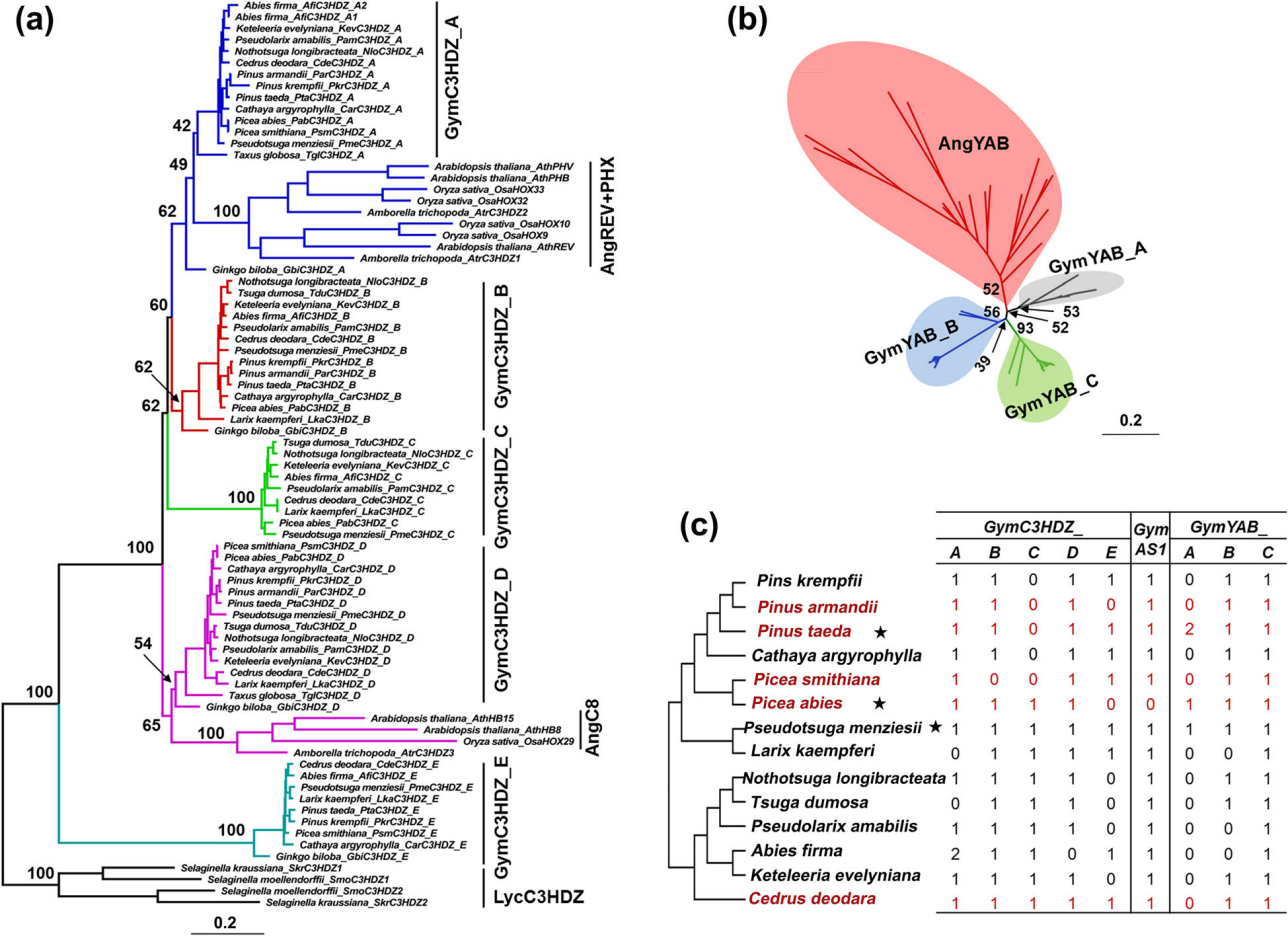
Maximum likelihood (ML) trees and repertoire of polarity genes. **a** Rooted phylogenetic tree of the *HDZIPIII* genes. **b** Unrooted trees of *YABBY* genes. Ang, angiosperm; Gym, gymnosperm; Lyc, lycophytes. Numbers on the branches indicate the bootstrap values. **c** Repertoire of polarity genes in Pinaceae lineages. Red colors indicate the species with needlelike leaves; Black stars indicate that data were collected from the genome databases

We searched three gene families involved in the establishment of leaf adaxial-abaxial polarity, and 56, 13 and 29 sequences of *HDZIPIII*, *AS1* and *YABBY*, respectively, were identified from 14 species representing all 11 genera of Pinaceae (Fig. 2). The phylogenetic analyses showed that homologs of the adaxial determinant *HDZIPIII* family in Pinaceae were divided into five distinct clades (GymC3HDZ_A to GymC3HDZ_E), in which GymC3HDZ_A was a sister to the angiosperm adaxial-patterning determinant AngREV+PHX clade and GymC3HDZ_D was a sister to the angiosperm AngC8 clade, whereas clades GymC3HDZ_B, GymC3HDZ_C, and GymC3HDZ_E were exclusive in gymnosperms (Fig. 2a). Besides, only one ortholog of another adaxial determinant *AS1* was identified in each Pinaceae species (Additional file 7: Figure S4). For the abaxial determinant *YABBY* gene family, an unrooted tree was built because this gene family was found only in seed plants [38, 39]. The results showed that gymnosperm sequences were distributed in three moderately supported clades (GymYAB_A, GymYAB_B and GymYAB_C), while the angiosperm sequences formed an independent clade (Fig. 2b).

It is interesting that most members in the three gene families (*HDZIPIII*, *AS1* and *YABBY*) occurred in almost all selected species, except the *GymC3HDZ_C* and *GymYAB_A* genes (Fig. 2c). *GymC3HDZ_C* genes were absent from the transcriptome databases of *Pinus*, *Cathaya* and *Picea*, and *GymYAB_A* genes were found only in the genome databases of *Pinus taeda*, *Picea abies* and *Pseudotsuga menziesii* (Fig. 2c).

### Spatiotemporal expression patterns of polarity genes in Pinaceae leaves

**Fig. 3:**
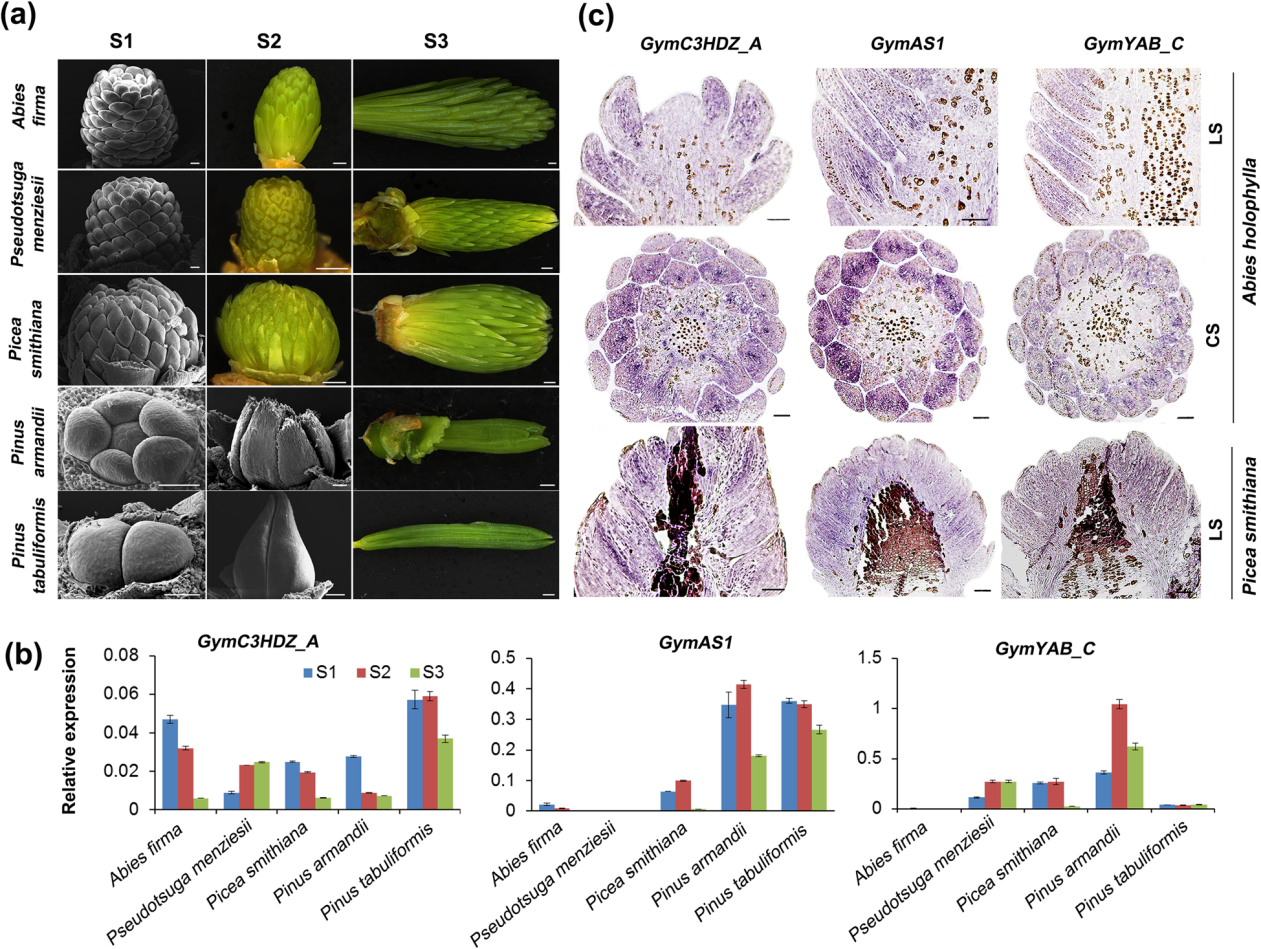
Spatiotemporal expression analyses of polarity genes. **a** Three developmental stages of Pinaceae leaves. Bars = 100 μm and bars = 1 mm for SEM and optical micrographs, respectively. **b** qPCR analyses of polarity genes in three leaf developmental stages. Error bars represent SD. **c** In situ localization of polarity genes in the vegetative tissues of *Abies holophylla* and *Picea smithiana*. LS, longitudinal sections; CS, cross sections. Bars, 100 μm

The qPCR analyses indicated that all four *HDZIPIII* family members (*GymC3HDZ_A* to *GymC3HDZ_D*) were expressed across the three stages of leaf development, particularly with prominent expression at the first two stages (Fig. 3 and Additional file 8: Figure S5). *GymC3HDZ_A*, *GymC3HDZ_C* and *GymC3HDZ_D* showed similar expression levels between the species with flattened and needlelike leaves, respectively (Fig. 3b and Additional file 8: Figure S5), whereas *GymC3HDZ_C* displayed higher expression mostly in the species with flattened leaves and a species with needlelike leaves, *Picea smithiana* (Additional file 8: Figure S5). In contrast to Gymnosperm *HDZIPIII* genes, another adaxial determinant gene, *GymAS1*, showed the strongest expression in the species with needlelike leaves, especially in the two pines (Fig. 3b). For the *YABBY* genes, the *GymYAB_C* homologs exhibited the same expression level in the two types of Pinaceae leaves (Fig. 3b), which was much higher than the expression level of the *GymYAB_B* homologs (Additional file 8: Figure S5).

According to phylogenetic and qPCR analyses, the expression patterns of the *GymC3HDZ_A*, *GymAS1* and *GymYAB_C* genes were further studied in *Abies holophylla* with flattened leaves and *Picea smithiana* with needlelike leaves by in situ hybridization. The expression signal of *GymC3HDZ_A* showed clear evidence of polar patterning that was restricted to the adaxial region and especially the vascular tissue in both species (Fig. 3c, the left column). The hybridization of *GymAS1* in the two species showed a strong expression signal through the central region between the adaxial and abaxial domains (Fig. 3c, the middle column), whereas *GymYAB_C* expression was detected only in the vascular tissues of the two species and slightly in the leaf margin of the cross section of *Abies holophylla* (Fig. 3c, the right column).

### Photosynthetic performance of the two types of Pinaceae leaves

**Fig. 4:**
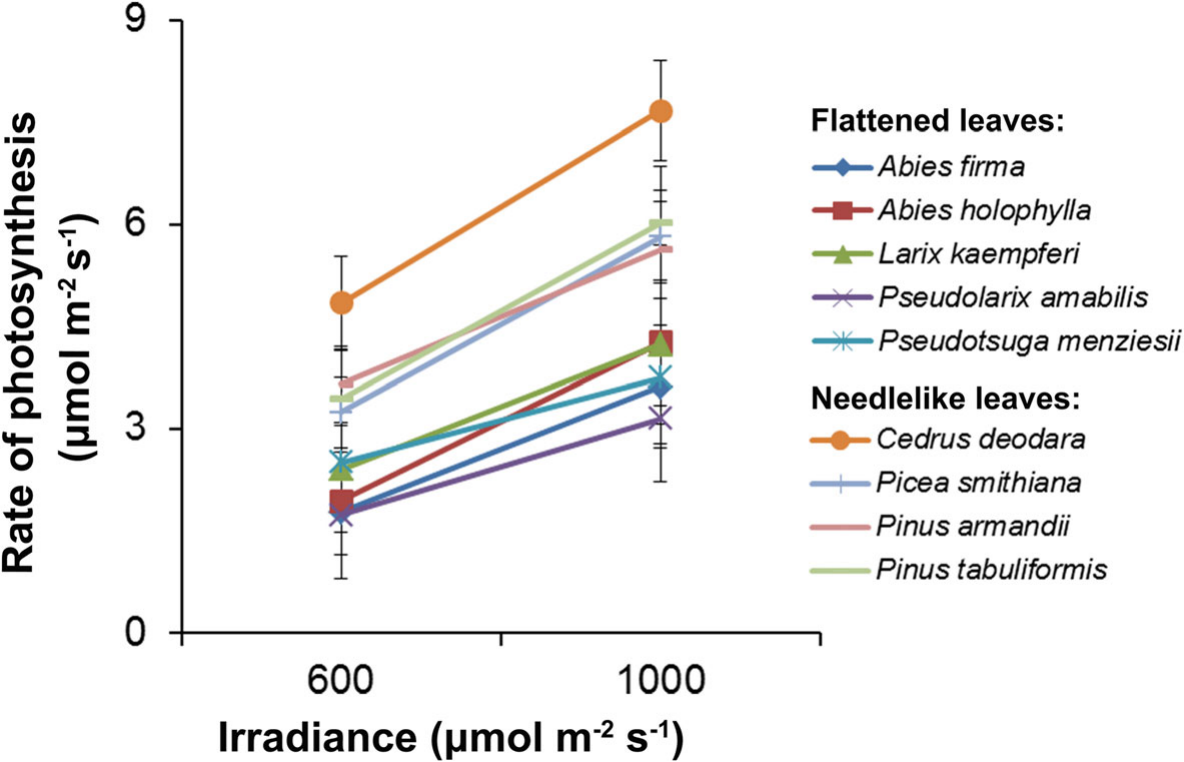
Rate of photosynthesis for needlelike and flattened leaves under two gradients of irradiance. All measurements were made on newly expanded leaves of trees growing in the same conditions in late August. Data are means of three replicates. Error bars represent SD

In the “common garden” conditions, the responses of photosynthesis to two PAR gradients in the representatives of the two types of Pinaceae leaves were examined. When the PAR was 600 mmol m^− 2^ s^− 1^, the net carbon assimilation of the species with flattened leaves (*Abies firma*, *Abies holophylla*, *Larix kaempferi*, *Pseudolarix amabilis* and *Pseudotsuga menziesii*) reached approximately 2 μmol m^− 2^ s^− 1^, while the species with needlelike leaves (*Cedrus deodara*, *Picea smithiana*, *Pinus armandii* and *Pinus tabuliformis*) exhibited a high photosynthetic capacity, i.e., 3–5 μmol m^− 2^ s^− 1^ (Fig. 4). Likewise, when facing a high light radiation of 1000 mmol m^− 2^ s^− 1^, the species with needlelike leaves also exhibited better photosynthetic capacity than the species with flattened leaves (Fig. 4). Similarly, the measurement of stomatal conductance and the transpiration rate showed relatively high values in the species with needlelike leaves (Additional file 9: Figure S6).

## Discussion

### A conserved adaxial-abaxial specifying mechanism exists in the development of different leaves in Pinaceae

Although the commonly known “needles” vary greatly in shape in Pinaceae, these leaves can be classified into two types, i.e., needlelike and flattened, based on the transverse WTR (Fig. 1a and Additional file 1: Figure S1). Structurally, the two types of leaves diverged in the differentiation and arrangement directions of the mesophyll tissues (Fig. 1b), but unexpectedly shared consistent spatial parallel patterning for the vasculatures (Fig. 1c), which usually implies dorsoventrality in the leaf [16, 31]. Therefore, the adaxial-abaxial polarity and the related specifying mechanism in the two types of leaves were studied by analyzing the Pinaceae homologs of the angiosperm polarity genes in the phylogenetic frame and spatiotemporal expression pattern in this study.

The *HDZIPIII*, *AS1* and *YABBY* genes play central roles in the antagonistic interactions of genes for the specification of leaf polarity in angiosperms [22, 25, 38]. In the present study, phylogenetic analyses grouped the Pinaceae adaxial determinants *HDZIPIII* and abaxial determinant *YABBY* into 5 and 3 clades, respectively (Fig. 2). Interestingly, these polarity genes did not show any obvious divergence in terms of allocation and number between the species with two different types of leaves (Fig. 2c). Congruent with the phylogeny of *HDZIPIII* in land plants [40, 41], our results indicate that all *HDZIPIII* homologs of seed plants form a highly supported monophyletic group, in which a gymnosperm clade is at the most basal position, and two sister clades between gymnosperm and angiosperm occur (Fig. 2a). This evidence, along with other previous studies [40–42], suggests that the *HDZIPIII* gene family diversified during seed plant evolution. One *AS1* member was identified in most of the Pinaceae species (Additional file 7: Figure S4), consistent with the fact that this gene exists as a single copy in most angiosperms [43–45]. Finet et al. (2016) [39] divided gymnosperm *YABBY* genes into four clades, of which three included Pinaceae genes. Consistently, in the present *YABBY* gene phylogeny, gymnosperm sequences are also distributed in three moderately supported clades, while the angiosperm sequences form an independent clade (Fig. 2b). Notably, the *YABBY* sequences generated from the transcriptome databases occur only in clades GymYAB_B and GymYAB_C (Fig. 2b), indicating the obligate function of these two clades for Pinaceae leaf development.

Increasing evidence from angiosperms supports that modifications of the expression patterns of polarity genes largely impact plant morphology, such as the trumpet leaves of the *HDZIPIII* double mutant and the narrow leaf blade of the *YABBY* triple mutants [20, 38, 46–49]. In the present study, according to the phylogenetic analysis (Fig. 2), *GymC3HDZ_A*, *GymAS1* and *GymYAB_C* were used in the qPCR and in situ hybridization experiments. In *Abies holophylla* and *Picea smithiana*, the in situ expression of *GymC3HDZ_A* displayed evident signals in the adaxial region and the provascular strands (Fig. 3c). The same expression pattern was also found in *Pseudotsuga menziesii* and *Ginkgo biloba* [40, 41] as well as in ferns, but not in lycophytes [42], which indicates that the role in leaf adaxial specification and provascular differentiation may only occur within the euphyllophytes [40–42]. The other adaxial determinant *AS1* homologs exhibited similar expression patterns constrained to the juxtaposition between the adaxial and abaxial domains (Fig. 3c), as is the case for its homolog in *Arabidopsis* [27]. In addition, the polar patterning of *GymYAB_C* was not found in the two types of leaves (Fig. 3c), which is similar to the results of a previous study on *Pseudotsuga menziesii* [39]. In addition to the in situ expression, the temporal expression by qPCR also indicates the critical functions of these polarity genes in Pinaceae leaf development (Fig. 3b and Additional file 8: Figure S5).

In conclusion, both the phylogenetic analyses and the spatiotemporal expression patterns indicate the existence of an evolutionarily conserved genetic network of polarity genes that confers adaxial-abaxial polarity in both the Pinaceae needlelike and flattened leaves.

### Pinaceae flattened and needlelike leaves have diverged for photosynthetic adaptation

As the basis of life on Earth, photosynthesis is mainly determined by three factors, i.e., stomata conductance, mesophyll conductance and photo/biochemistry [35, 50, 51], all of which are closely linked to leaf morphology and structure. Along the evolution of land plants, leaf morphology and structure have changed greatly in parallel with the progressively increasing maximum photosynthesis rates, from the lowest values in bryophytes to the highest values in angiosperms [52, 53]. In the present study, we found that the species with needlelike leaves generally exhibited better photosynthetic performance than the species with flattened leaves (Fig. 4 and and Additional file 9: Figure S6). Similar results were found in other studies. For instance, because of the difference in mesophyll conductance, the drought-adapted *Abies pinsapo* with needlelike amphistomatous leaves showed a higher photosynthetic rate than *Abies alba* with flattened, hypostomatous leaves in the mesic habitat [54]. Brodribb et al. (2007) [37] also found a higher photosynthetic capacity in needle-leaved *Pinus* species than in flat-leaved Podocarpaceae species, supporting the hypothesis that the length of the hydraulic pathway of the post-vein traverse through the mesophyll is negatively correlated with hydraulic conductivity as well as the CO_2_ assimilation rate. A recent study concluded that in gymnosperms, photosynthesis was mainly colimited by stomatal and mesophyll conductance [50]. Therefore, the divergence in photosynthesis of Pinaceae flattened and needlelike leaves could largely be attributed to the leaf structure that directly affects CO_2_ conductance.

In environments with the greatest sun exposure, especially those with concurrent stresses, such as water limitations, the leaf form appears to be more cylindrical with the radial, cross-section geometry [55]. Likely, the relatively dry habitats of the Northern Hemisphere shaped the needlelike, linear or scale-like leaves in Pinaceae and most Cupressaceae species that are characteristic of xeric habitats, while the warm and wet tropical forests in the Southern Hemisphere are more favorable for the development of bilaterally flattened foliage in Araucariaceae and Podocarpaceae [3, 56–58]. In addition, a phylogenetically based analysis suggested that Cupressaceae evolved from ancestors living in moist habitats with bilaterally flattened foliage to drought-resistant crown clades with appressed, imbricated leaves, accompanied by a reduced photosynthetic capacity because of the decreased xylem-specific conductivity [6, 59]. Therefore, the different leaf morphologies were accompanied by divergent photosynthetic adaptation [5]. In Pinaceae, species with different types of leaves appear to have diverged in ecological niches. For instance, the genera *Pinus*, *Picea* and *Cedrus* with needlelike leaves are more suited to drought habitats than the genera with flattened leaves, such as *Keteleeria* and *Tsuga*, which inhabit warm temperate and subtropical forests [57]. Similarly, a recent study indicated that spruce with quadrangular leaves (sect. *Picea*) tend to be distributed in dry habitats, while those with flattened or broad triangular leaves (sect. *Omorica* and sect. *Casicta*) occur mainly in wet climates [60]. Based on the present study, the structure of dense mesophyll tissue, an almost multifaceted stomatal line and a high photosynthetic rate could help the species of Pinaceae with needlelike leaves live in drought-prone and sunny habitats (Fig. 1b and Fig. 4). In contrast, the porous mesophyll tissue, particularly the uniform palisade cells and relatively low photosynthetic rate, could allow the species with flattened leaves to survive in the humid and shady environment (Fig. 1b and Fig. 4). More studies related to the physiological and ecological attributes are needed to better understand the adaptive evolution of Pinaceae leaves.

## Conclusions

Leaf developmental mechanisms have been studied extensively in angiosperms, but rarely in gymnosperms due to their long generation times and large genome sizes. In this study, based on a detailed morpho-anatomical study of leaves from all 11 Pinaceae genera, the expression patterns of adaxial-abaxial polarity genes were investigated in the delimited needlelike and flattened leaves of this family. We found that the two types of leaves share conserved spatial patterning of vasculatures and genetic networks for adaxial-abaxial polarity, although the differentiation and arrangement direction of the mesophyll tissues are different. In addition, the two types of leaves have diverged for photosynthetic adaptation with the needlelike leaves exhibiting better photosynthetic performance than the flattened leaves. Our study indicates the existence of an evolutionarily conserved genetic network of polarity genes that confers adaxial-abaxial polarity in both the needlelike and flattened leaves of Pinaceae.

## Methods

### Plant materials

Because leaf morphology is generally conserved within most genera of Pinaceae, a total of 16 species representing all 11 genera of Pinaceae were sampled (Additional file 10: Table S1). Sampling permissions were obtained from the corresponding botanical gardens and natural reserves. Sample identifications were performed according to the species signboard in the botanical gardens as well as and the records of Flora of China and some voucher specimens were deposited in our lab. More than one species was selected from both *Picea* and *Pinus* because both needlelike and flattened leaves occur in these two genera. In addition, two species of *Abies* were used to test the morphological variation within the genus, and two were studied from *Tsuga* due to the shortage of fresh materials. Each species was represented by 1–3 individuals. First, all samples, with the exception of *Pinus armandii*, were used to observe the morphology and anatomy by optical microscopy and scanning electron microscopy (SEM). Second, 5 species belonging to 4 genera representing flattened and needlelike leaves were used to analyze the expression of adaxial-abaxial polarity genes by qPCR. Then, *Abies holophylla* with flattened leaves and *Picea smithiana* with needlelike leaves were used in the in situ hybridization. Finally, based on the “common garden” conditions, 9 species representing 7 genera cultivated at the Institute of Botany, Chinese Academy of Sciences (IBCAS), were selected to measure the photosynthetic capacity.

### Morphological and anatomical observations

Current-year fresh mature leaves were used to observe the morphology and anatomy under a stereomicroscope (M205 C, Leica Camera AG, Germany). The width/thickness ratio (WTR) of leaf cross section was introduced to classify leaves of different types. ImageJ software was used to measure the width and thickness of the photographed cross sections.

SEM (S-4800 FESEM, Hitachi, Japan) was used to magnify and clearly observe the mesophyll structure and leaf bud morphology. Fresh mature leaves and leaf buds of different developmental stages were collected and immediately fixed in FAA (Formalin-Acetic-Alcohol). The FAA solution was infiltrated for up to 30 min under vacuum and then stored at 4 °C. Samples were dehydrated in a water-ethanol series and then put in a CO_2_ critical-point dryer. The dried materials were fixed on a small round table to be sputter-coated with gold. After that, the materials were examined with SEM.

In addition, because some sclereids were found in *Pinus krempfii*, *Pseudotsuga menziesii* and *Nothotsuga longibracteata* (see results), high-resolution three-dimensional (3D) images of the internal and geometric structures of the leaves of these three species were reconstructed using a micro-computed tomography (micro-CT) scanner (Bruker SkyScan 1172, Bruker Corp., USA). The pretreatment of materials followed the above fixing and drying procedures used for the SEM observations. The dried materials were fixed on a cylindrical bench, which could rotate between the fixed X-ray source and the detector. The two-dimensional tomographic images were produced by computer-processed X-rays, and the 3D images were generated by subsequent digital geometry processing. NRecon v.1.6 software and the embedded flight recorder were used to reconstruct the 3D structure and convert the slices into animated movies by rotating, translating or scaling the models.

### Data collection and phylogenetic reconstruction of polarity genes

To confirm whether adaxial-abaxial polarity exists in different Pinaceae leaves, the homologs of *HDZIPIII*, *AS1* and *YABBY* genes that play key roles in adaxial-abaxial polarity establishment were studied. *HDZIPIII* and *AS1* represent the adaxial determinants [20, 22], and the *YABBY* genes are involved not only in the abaxial development but also in leaf expanding [21], which is important for understanding the developmental mechanism of the flattened leaves in Pinaceae. First, the polarity genes were identified from the available transcriptome databases of the 11 Pinaceae genera by tBLASTn, using the corresponding genes of *Arabidopsis thaliana* as queries. These transcriptomes were previously sequenced by our lab with leaf bud and/or mature leaves as the materials [11]. In addition, the genome databases of *Picea abies*, *Pinus taeda*, *Pseudotsuga menziesii*, *Ginkgo biloba*, *Amborella trichopoda* and *Selaginella moellendorffii* were also used for gene searches. Some previously identified *HDZIPIII* and *YABBY* sequences of *Ginkgo biloba*, *Taxus globosa*, *Pseudotsuga menziesii*, *Pinus taeda*, *Selaginella kraussiana* and *Selaginella moellendorffii* were also downloaded from GenBank [39, 40]. In addition, homologous sequences from three model angiosperm species, including *Arabidopsis thaliana*, *Oryza sativa* and *Amborella trichopoda*, were added to the analysis [40, 61]. The accession numbers of all the sequences used in this study are shown in Additional file 11: Table S2.

To reconstruct the phylogenetic trees, the coding regions of the polarity genes were first aligned with MAFFT [62] and manually adjusted in BIOEDIT [63]. To delete poorly aligned positions, the aligned CDS sequences were processed through ZORRO [64] with a threshold of 4. Then, nucleotide substitution saturation was tested for each codon position using DAMBE 5 [65], which suggested that all three codon positions were not saturated for these genes. Therefore, the CDS datasets were used for the following analyses. Maximum likelihood (ML) searches were performed using RAxML v.8.1 [66] under the GTRGAMMA model. One hundred bootstrap replicates were conducted for support estimation.

### RNA extraction and qPCR analysis

To explore the expression patterns of the adaxial-abaxial polarity genes, we selected five representative species (*Abies firma*, *Pseudotsuga menziesii*, *Pinus armandii*, *Pinus tabuliformis* and *Picea smithiana*) of the flattened and needlelike leaves to perform qPCR analysis. *AS1* and some key members of the *HDZIPIII* and *YABBY* gene families were selected for analysis based on previous studies [22, 39–41] and the present phylogenetic reconstruction (Fig. 2). For *HDZIPIII*, all clades of the seed plant *HDZIPIII* genes, with the exception of GymC3HDZ_E, were selected (Fig. 2a). For *YABBY*, only *GymYAB_B* and *GymYAB_C* genes were identified in the transcriptome sequences (Fig. 2b), and therefore, these two clades were selected. Primers were designed based on sequence alignments. In the developmental process of the Pinaceae leaves, three key stages (S1 to S3) were specifically considered, from the stage of leaf primordia (S1 in Fig. 3a) to the stage of acquiring the initial leaf morphology (S2 in Fig. 3a) and finally the stage of middle-aged leaves (S3 in Fig. 3a). Fresh materials were dissected and immediately frozen in liquid nitrogen. Total RNA was extracted with an RNAprep Pure Plant Plus Kit (polysaccharides & polyphenolics-rich) (DP441, TIANGEN, China).

Approximately 2 μg of total RNA was reverse transcribed into cDNA using an RT-PCR kit (KR116, TIANGEN, China). The qPCR analysis was performed on a StepOnePlus Real-Time PCR System (Life Technologies Corp., USA) with the TB Green® Premix Ex Taq™ reagent (RR420A, TAKARA, Japan). Quantification was performed using the 2^-△CT^ method and the data were normalized based on the quantity of the reference gene, *ACTIN*. The dissociation curves were analyzed in all amplifications.

### In situ hybridization

To test whether the polarity genes also present the adaxial-abaxial expression pattern in the distinct Pinaceae leaves, the samples of *Abies holophylla* and *Picea smithiana* were used for in situ hybridization. Leaf primordial tissues were fixed in FAA or paraformaldehyde (PFA) for 12 h and then dehydrated in a graded ethanol series on ice. The dehydrated samples in 100% ethanol were replaced with a graded xylene series and finally embedded in paraplast (P3683, SIGMA). The gene-specific probes labeled by digoxigenin were generated according to the manufacturer’s instructions for a DIG Northern Starter Kit (No. 12039672910, Roche). The probe length was usually approximately 200 bp. The whole procedure of in situ hybridization was carried out as described by Brewer [67] with some small modifications. Slides were examined and photographed on a Leica DM6 B microscope.

### Measurement of photosynthetic capacity

The measurement of photosynthetic capacity was conducted on 8 species growing in IBCAS. Approximately 3–5 branches were cut from 2 to 3 trees of each species in late August and end-soaked in water before being measured in the lab. All measurements were collected from newly expanded leaves (approximately 3–6 leaves each time) with a portable gas-exchange and fluorescence system (GFS-3000, Walz, Germany). The environmental parameters were set as follows: an air-flow of 400 mmol s^− 1^ through the chamber, a leaf temperature of 30 °C, a CO_2_ concentration of 380 μmol mol^− 1^, and two photosynthetically active radiation (PAR) gradients of 600 and 1000 mmol m^− 2^ s^− 1^. The measured leaves were then used to calculate the projected leaf area.

## Supplementary information

**Additional file 1: Figure S1.** Diverse morphologies of Pinaceae leaves. (a) *Abies firma*. (b) *Abies holophylla*. (c) *Cathaya argyrophylla*. (d) *Keteleeria davidiana*. (e) *Larix kaempferi*. (f) *Nothotsuga longibracteata*. (g) *Pseudolarix amabilis*. (h) *Pseudotsuga menziesii*. (i) *Tsuga chinensis*. (j) *Cedrus deodara*. (k) *Picea smithiana*. (l) *Picea brachytyla* var. *complanata*. (m) *Pinus krempfii*. (n) *Pinus tabuliformis*. Ads, adaxial side; Abs, abaxial side. Bars, 1 mm.

**Additional file 2: Figure S2.** SEM micrographs of sclereids from leaves of *Pinus krempfii* (a), *Pseudotsuga menziesii* (b) and *Nothotsuga longibracteata* (c)*.* Red arrows indicate the sclereids. Bars, 200 μm.

**Additional file 3: Video S1.** Sclereids of *Pinus krempfii* shown in Additional file 2: Figure S2a.

**Additional file 4: Video S2.** Sclereids of *Pseudotsuga menziesii* shown in Additional file 2: Figure S2b.

**Additional file 5: Video S3.** Sclereids of *Nothotsuga longibracteata* shown in Additional file 2: Figure S2c.

**Additional file 6: Figure S3.** Phloroglucinol staining of xylem in the needlelike leaves of *Cedrus deodara* (a), *Picea smithiana* (b), *Pinus armandii* (c) and *Pinus tabuliformis* (d). xy, xylem; ph, phloem. Bars, 100 μm.

**Additional file 7: Figure S4.** Sequence alignment of AS1 from seed plants.

**Additional file 8: Figure S5.** qPCR analyses of polarity genes in three leaf developmental stages of the flattened- and needlelike-leaved species.

**Additional file 9: Figure S6.** Stomatal conductance and transpiration rate for needlelike and flattened leaves under two gradients of irradiance. Data are means of three replicates.

**Additional file 10: Table S1.** Samples used in this study.

**Additional file 11: Table S2.** The accession numbers of all sequences used in this study.

## Data Availability

The new sequences identified in this study have been submitted to GenBank, with accession numbers shown in Additional file 2: Table S2.

## Abbreviations

C3HDZ: Class III HD-ZIP
YAB: YABBY
SEM: Scanning electron microscopy
IBCAS: Institute of Botany, Chinese Academy of Sciences
micro-CT: microcomputed tomography
FAA: Formalin-Acetic-Alcohol
PFA: Paraformaldehyde
ML: Maximum likelihood
PAR: Photosynthetically active radiation
