# Supplementary figures and images for: The flattened and needlelike leaves of the pine family (Pinaceae) share a conserved genetic network for adaxial-abaxial polarity but have diverged for photosynthetic adaptation
Source: BMC Evol Biol. 2020 Oct 7;20:131. doi: 10.1186/s12862-020-01694-5 (PMC7542717; doi:10.1186/s12862-020-01694-5)

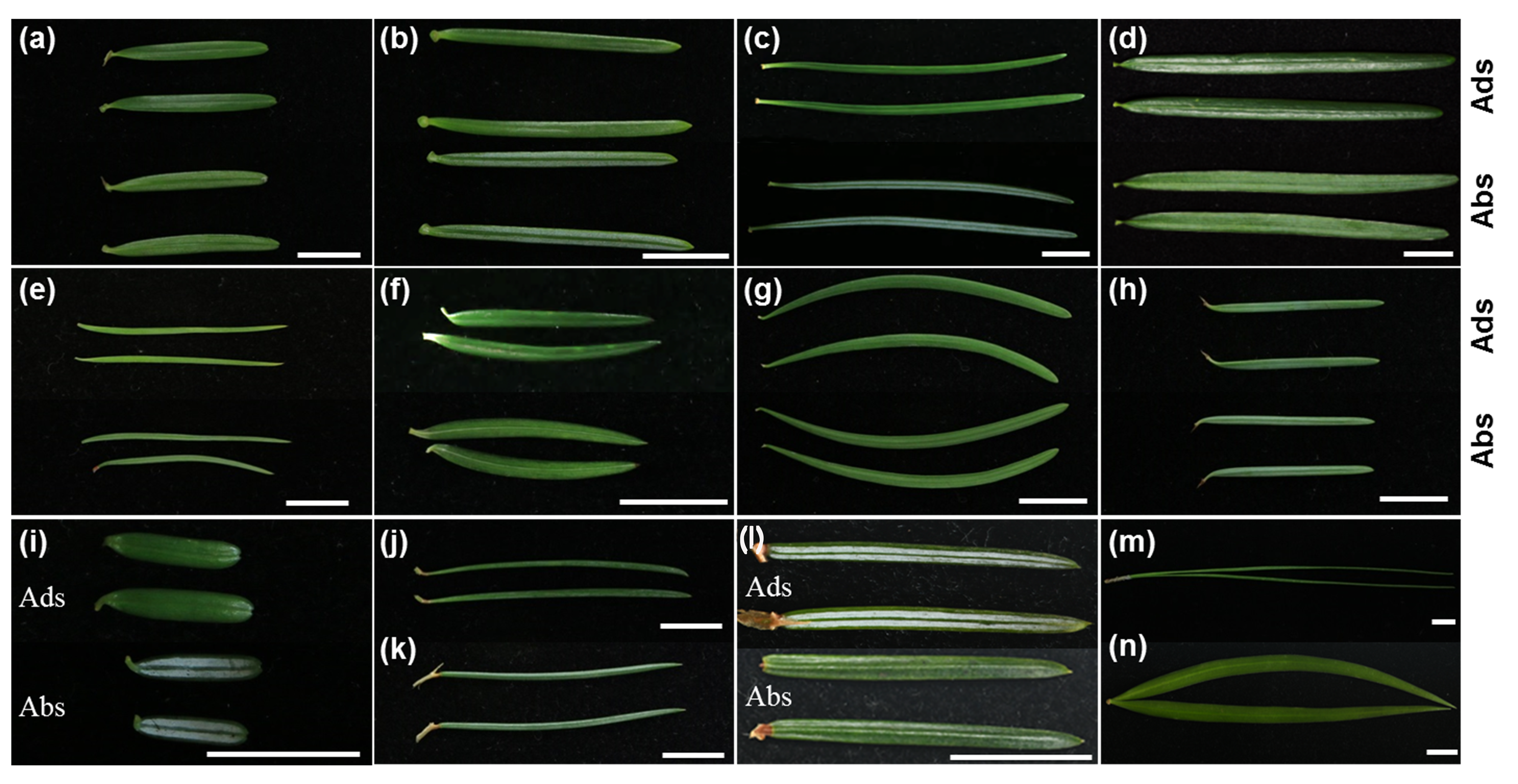

Supplement: Supplementary file 1 — Additional file 1: Figure S1. Diverse morphologies of Pinaceae leaves. (a) Abies firma. (b) Abies holophylla. (c) Cathaya argyrophylla. (d) Keteleeria davidiana. (e) Larix kaempferi. (f) Nothotsuga longibracteata. (g) Pseudolarix amabilis. (h) Pseudotsuga menziesii. (i) Tsuga chinensis. (j) Cedrus deodara. (k) Picea smithiana. (l) Picea brachytyla var. complanata. (m) Pinus krempfii. (n) Pinus tabuliformis. Ads, adaxial side; Abs, abaxial side. Bars, 1 mm. [file 12862_2020_1694_MOESM1_ESM.tif]

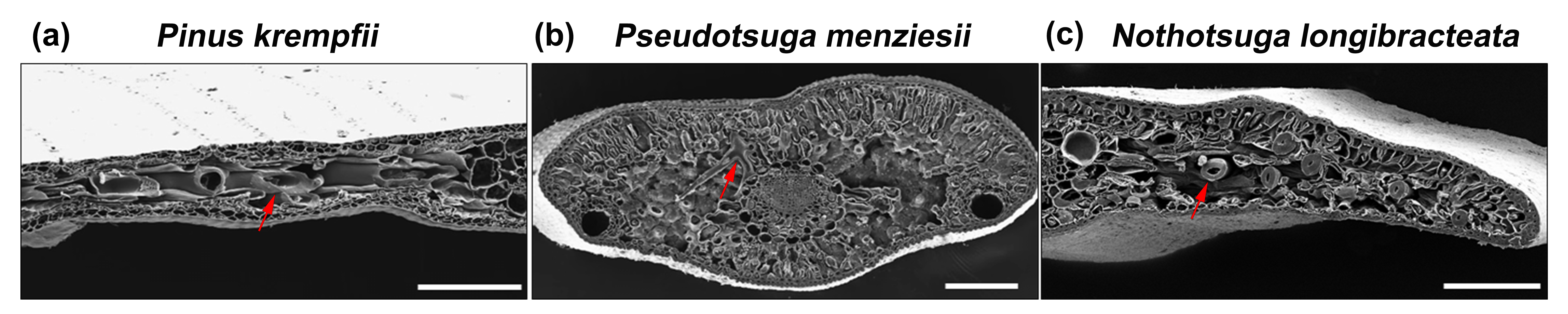

Supplement: Supplementary file 2 — Additional file 2: Figure S2. SEM micrographs of sclereids from leaves of Pinus krempfii (a), Pseudotsuga menziesii (b) and Nothotsuga longibracteata (c). Red arrows indicate the sclereids. Bars, 200 μm. [file 12862_2020_1694_MOESM2_ESM.tif]

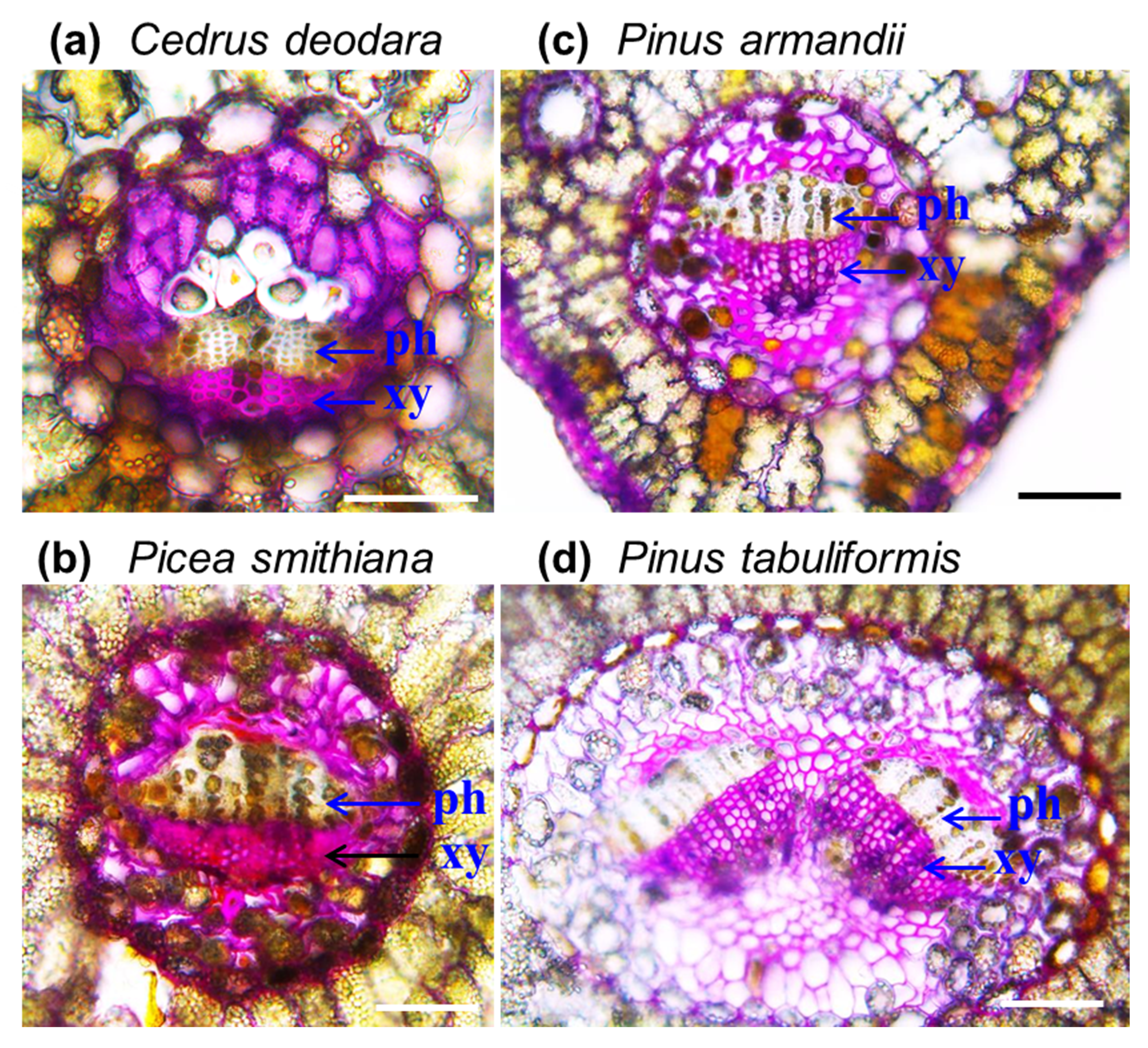

Supplement: Supplementary file 6 — Additional file 6: Figure S3. Phloroglucinol staining of xylem in the needlelike leaves of Cedrus deodara (a), Picea smithiana (b), Pinus armandii (c) and Pinus tabuliformis (d). xy, xylem; ph, phloem. Bars, 100 μm. [file 12862_2020_1694_MOESM6_ESM.tif]

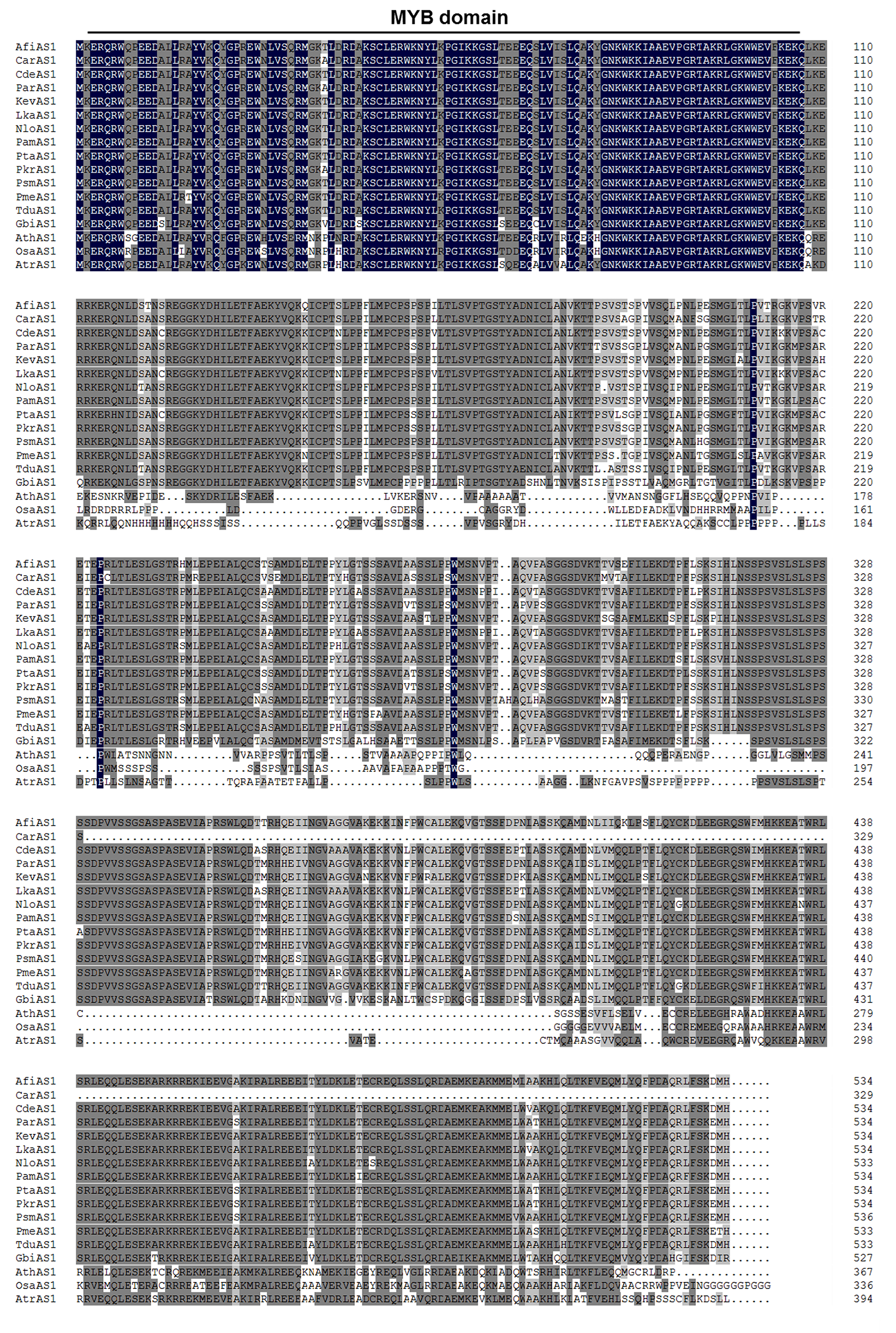

Supplement: Supplementary file 7 — Additional file 7: Figure S4. Sequence alignment of AS1 from seed plants. [file 12862_2020_1694_MOESM7_ESM.tif]

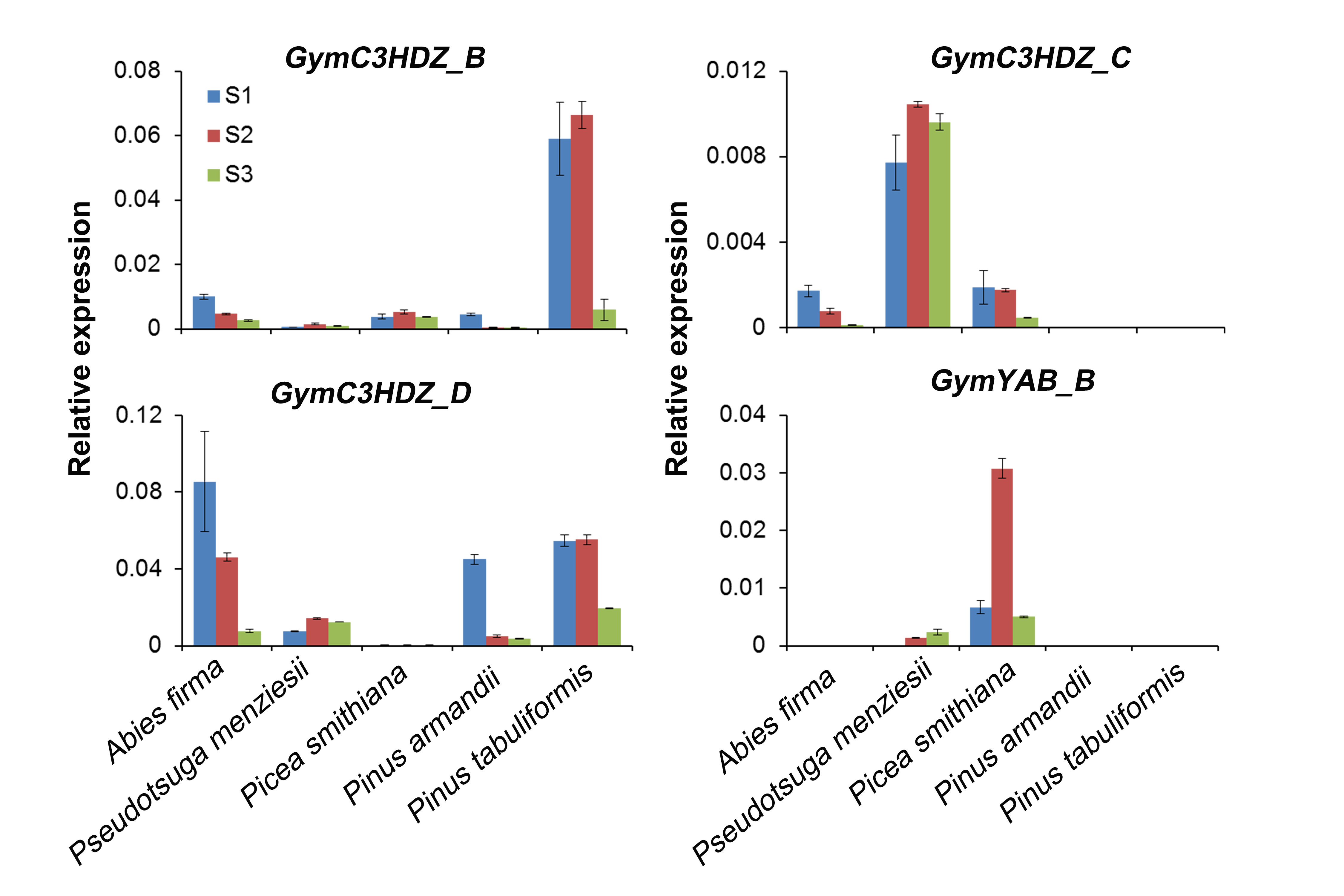

Supplement: Supplementary file 8 — Additional file 8: Figure S5. qPCR analyses of polarity genes in three leaf developmental stages of the flattened- and needlelike-leaved species. [file 12862_2020_1694_MOESM8_ESM.tif]

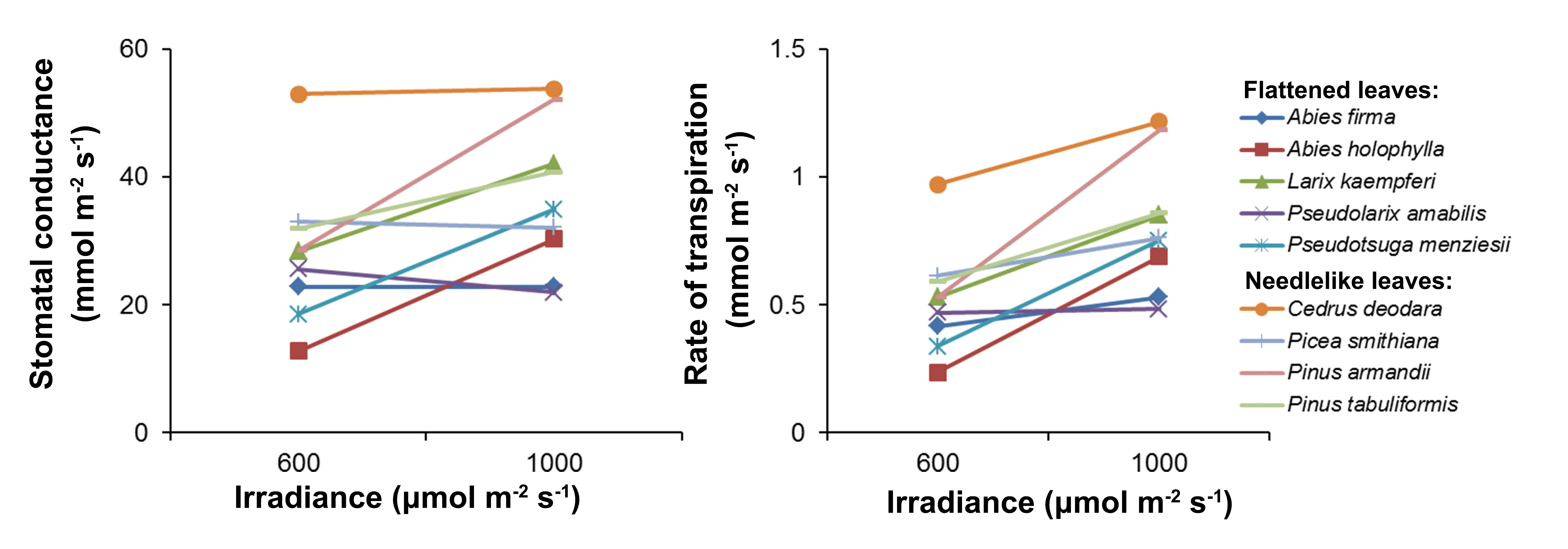

Supplement: Supplementary file 9 — Additional file 9: Figure S6. Stomatal conductance and transpiration rate for needlelike and flattened leaves under two gradients of irradiance. Data are means of three replicates. [file 12862_2020_1694_MOESM9_ESM.tif]
